# Codon usage bias reveals genomic adaptations to environmental conditions in an acidophilic consortium

**DOI:** 10.1371/journal.pone.0195869

**Published:** 2018-05-09

**Authors:** Andrew Hart, María Paz Cortés, Mauricio Latorre, Servet Martinez

**Affiliations:** 1 UMI 2071 CNRS-UCHILE, Facultad de Ciencias Físicas y Matemáticas, Centro de Modelamiento Matemático, Universidad de Chile, Casilla 170, Correo 3, Santiago, Chile; 2 Mathomics, Centro de Modelamiento Matemático, Universidad de Chile, Santiago, Chile; 3 Fondap-Center of Genome Regulation, Facultad de Ciencias, Universidad de Chile, Santiago, Chile; 4 Laboratorio de Bioinformática y Expresión Génica, INTA, Universidad de Chile, Macul, Santiago, Chile; 5 Universidad de O'Higgins, Instituto de Ciencias de la Ingeniería, Rancagua, Chile; 6 Departamento de Ingeniería Matemática, UMI 2071 CNRS-UCHILE, Facultad de Ciencias Físicas y Matemáticas, Centro de Modelamiento Matemático, Universidad de Chile, Casilla 170, Correo 3, Santiago, Chile; Oklahoma State University, UNITED STATES

## Abstract

The analysis of codon usage bias has been widely used to characterize different communities of microorganisms. In this context, the aim of this work was to study the codon usage bias in a natural consortium of five acidophilic bacteria used for biomining. The codon usage bias of the consortium was contrasted with genes from an alternative collection of acidophilic reference strains and metagenome samples. Results indicate that acidophilic bacteria preferentially have low codon usage bias, consistent with both their capacity to live in a wide range of habitats and their slow growth rate, a characteristic probably acquired independently from their phylogenetic relationships. In addition, the analysis showed significant differences in the unique sets of genes from the autotrophic species of the consortium in relation to other acidophilic organisms, principally in genes which code for proteins involved in metal and oxidative stress resistance. The lower values of codon usage bias obtained in this unique set of genes suggest higher transcriptional adaptation to living in extreme conditions, which was probably acquired as a measure for resisting the elevated metal conditions present in the mine.

## Introduction

A total of 61 sense codons translate into 20 different amino acids, which is known as the redundancy of the genetic code or degeneracy of codons. Codon usage bias (CUB) refers to differences in the relative frequencies of synonymous codons within a coding sequence, differences which have been correlated with functional and adaptive properties [1–3]. The absence of CUB means that synonymous codons are used randomly without preference to code for their corresponding amino acids. A coding sequence is said to have low or weak CUB when synonymous codons are employed in a mostly random way. In contrast, high or strong CUB ensues when synonymous codons are used in a preferential manner to code for amino acids, the most extreme case being when exactly one codon is used to represent each amino acid.

The analysis of CUB has been used to characterize both specific and general properties of genes from communities of microorganisms [4]. Botzman *et al* determined an association between the lifestyles of several prokaryotic organisms and variations in their CUB [5]. Their results indicated that species living in a wide range of habitats have low CUB, which is consistent with the need to adapt to different environments. In addition, results also suggest that species may more readily adjust to metabolic variability by maintaining low CUB.

Bacteria which use a small subset of optimal codons (high CUB) also present fast growing rates [6], supporting the idea that optimization of the translation machinery is correlated with the maximization of growth rate. Complementing these studies, the analysis of 11 sequenced microbial samples showed that organisms living in the same ecological niche share a common preference for CUB, regardless of their phylogenetic diversity [7]. Such evidence highlights the importance of analyzing CUB in order to characterize bacterial communities, studies not hitherto addressed in acidophilic species.

Acidophilic bacteria are characterized by their survival under low pH and high concentration of metal cations. They are some of the most studied microorganisms living in extreme environments and are widely employed for the recovery of precious metals from mineral ores. During the process of extracting metal ions from different ores or concentrates, several microbial species work in concert in order to convert insoluble metal sulfides into water-soluble metal sulfates [8,9]. Currently, it is known that biomining communities of extremophile microorganisms that act in a coordinated manner are able to achieve higher levels of performance in metal extraction processes [10–12].

Several efforts have been made to isolate and characterize bacterial species and communities from differing extreme environmental sites [13–18]. At the molecular level, most criteria have been focused on identifying and quantifying particular components of each bacterium, such as proteins involved in iron/sulfur oxidation, metal resistance and biofilm formation [19–21]. While this strategy is able to suggest direct correlations between some of these components and a greater capacity for mineral bioleaching, only a few global-scale studies with the objective of imputing genomic advantages or common properties to such communities have been undertaken.

The first study to shed light on community gene structures in a mine environment was presented in 2004 by Tyson et al. [22]. Performing a metagenomic analysis, it was determined that a microbial community inhabiting acid mine drainage combines carbon and nitrogen fixation pathways in order to survive in such an extreme environment. With the aim of investigating genomic properties of a bacterial community from an industrially bioleached mine, a metagenome analysis of a surface layer of low grade copper tailings was recently undertaken at the Dexing Copper Mine in China [23,24]. The results illustrated that metal cation transport and DNA repair are highly represented processes inside the community, highlighting the presence of *Acidithiobacillus* and *Acidiphilium* species. In addition, the afore-mentioned studies provide a complete dataset of genes from acidophilic bacterial species, opening the possibility to study, characterize and classify extreme communities of microorganisms according to their CUB.

Recently, a consortium of five natural copper-bioleaching acidophilic bacteria was presented [25]. The consortium is made up of the bacteria *Acidithiobacillus thiooxidans* Licanantay, *Acidiphilium multivorum* Yenapatur, *Leptospirillum ferriphilum* Pañiwe, *Acidithiobacillus ferrooxidans* Wenelen and *Sulfobacillus thermosulfidooxidans* Cutipay, which were directly isolated from copper mines and selected based on their high capacity to solubilize copper and resist high concentrations of metal cations. In addition, this consortium is currently employed in a fully operational biotechnology system at CODELCO, Radomiro Tomic Division (Patent Registration No. CL 48319, Antofagasta, Chile).

In order to determine if this natural consortium of extreme acidophilic bacteria exhibits any particular genomic advantages, the CUB of genes belonging to the consortium were contrasted with: i) an acidophilic biomining consortium (metagenomic data) from a surface layer of low grade copper tailings (Dexing Copper Mine, China), ii) an alternative (non-consortium) collection of reference acidophilic bacterial strains (which were independently isolated from different geographic locations around the world) and iii) a global bacterial CUB profile generated from a set of reference genes compiled from the 2014 COG database. Considering the particular niches they inhabit, the consortium, non-consortium and metagenomic data were compared with a view towards determining if discrepancies in patterns of CUB are correlated with the extreme environments they inhabit.

## Materials and methods

### Genome sequences

The gene and protein sequences for *Sulfobacillus thermosulfidooxidans* strain Cutipay, *Acidithiobacillus thiooxidans* strain Licanantay, *Acidiphilum multivorum* strain Yenapatur, *Leptospirillum ferriphilum* strain Pañiwue and *Acidithiobacillus ferrooxidans* strain Wenelen, which constitute the bacterial species belonging to a Chilean biomining consortium, were previously described [25–27]. Their sequences are available at http://biominingdb.cmm.uchile.cl/genomes/. Sequences for the group of non-consortium species were downloaded from the NCBI. This group consists of *Acidimicrobium ferrooxidans* DSM 10331, *Acidiphilium cryptum* JF-5, *Acidiphilium multivorum* AIU301, *Acidiphilium sp*. PM, *Acidithiobacillus caldus* ATCC 51756, *At*. *caldus* SM-1, *At*. *ferrivorans* SS3, *At*. *ferrooxidans* ATCC 23270, *At*. *ferrooxidans* ATCC 53993, *At*. *thiooxidans* ATCC19377, *Desulfosporosinus acidiphilus* DSM 22704, *Leptospirillum ferriphilum* ML-04, *L*. *ferrooxidans* C2-3, *Sulfobacillus acidophilus* TPY, *Sb*. *thermosulfidooxidans* DSM 9293, *Sb*. *thermosulfidooxidans* CBAR-13, *Thiomonas intermedia* K12 and *Thiomonas sp* 3As (Accessions NC_013124.1; NC_009484.1, NC_009467.1-NC_009474.1; NC_015186.1; NZ_AFPR01000001.1-NZ_AFPR01000627.1; NZ_CP005986.1-NZ_CP005989.1; NC_015850.1-NC_015854.1; NC_015942.1; NC_011761.1; NC_011206.1; NZ_AFOH00000000.1; NC_018066.1-NC_018068.1; NC_018649.1; NC_017094.1; NC_015757.1; FWWY01000001.1-FWWY01000002.1; NZ_LGRO00000000.1; NC_014153.1-NC_014155.1 and NC_014144.1-NC_014145.1 respectively)[28–40]. Metagenomics data from a bioleaching heap sample presented in Zhang, X. *et al.[23]* were downloaded from the MG-RAST repository (Accession 4664533.3)[41]. Putative gene sequences in this sample and their taxonomic categories were also obtained from this repository (328571 sequences in total with taxonomic assignments made through similarity searches against RefSeq proteins with cutoffs: 15bp alignment length; e-5 e-value; 60% identity).

### Orthologous genes

Each bacterium in the consortium group was paired with one of the same species in the non-consortium group: *Sb*. *thermosulfidooxidans* Cutipay and CBAR-13; *At*. *thiooxidans* Licanantay and ATCC19377; *A*. *multivorum* Yenapatur and AIU301; *L*. *ferriphilum* Pañiwue and ML-04; and *At*. *ferroxidans* Wenelen and ATCC23270. For each pair, orthologous genes were calculated using both ORTHOMCL v1.4 [42] and Inparanoid v4.1 [43]. Only gene pairs predicted as orthologous by both tools were kept.

### COG category assignment

COG categories for all groups were assigned based on a protein BLAST search against the 2014 COG database with e-value and identity cutoffs of 1e^-5^ and 40% respectively. In the case of protein sequences from the metagenomic sample, only those with a length of at least 90% of the hit length were considered (35000 sequences in total). This set was taken as the metagenome group.

### COG database to gene database

A gene sequence database based on the 2014 COG protein database was generated for use as a reference set of non-lifestyle-specific genes. GenBank accessions for proteins in the COG database were retrieved from NCBI (ftp://ftp.ncbi.nih.gov/pub/COG/COG2014/data). Using those accession codes the associated genome gbk files were downloaded from NCBI. Finally, gene sequences corresponding to COG proteins were retrieved from these files and a gene sequence database was constructed containing a total of 1,737,559 DNA sequences.

### Kullback–Leibler codon information bias (CIB)

We use the Kullback-Leibler codon information bias (CIB) defined in [44] as a way of quantifying the use of synonymous codons in genes relative to the reference scenario in which each synonymous codon is used equally often to code for its corresponding amino acid (see [45], for an examination of various measures of CUB based on other principles). More explicitly, CIB is a measure of codon usage bias based on information theoretic concepts, namely entropy, which takes account of how amino acids are distributed. As such, CIB is a natural and intuitively appealing quantity for measuring the departure of a coding sequence from equal usage of synonymous codons (details in S1 Appendix). CIB is zero if and only if the codons that code for each amino acid are used equally often to represent that amino acid, that is, there is unbiased synonymous codon usage. It attains its maximum value, which is determined by the relative frequencies of all the amino acids, precisely when each amino acid is represented by exactly one codon. Small values of CIB correspond to low (less selective or weak) codon usage bias while larger values of CIB correspond to a greater concentration of the codon relative frequencies on fewer codons (stronger or more selective codon usage bias). For this study, CIB was rescaled to have a value in the range 0–1.

### Data analysis and statistical tools

The value of CIB was computed for every gene annotated for all bacterial species under consideration and for every putative gene belonging to the metagenomic sample. In addition, CIB was calculated for the 1,737,559 genes in the gene database derived from the COG database. This constitutes 97.3% of the 1,785,722 genes listed in the 2014 COG database. The remaining 2.7% of genes in the COG database were excluded from this study as it was not technically possible to recover the coding sequences needed for calculating the codon relative frequencies; the computation of CIB requires both the amino acid relative frequencies and the codon relative frequencies.

Differences in the pattern of CIB were analyzed between strains of the same organism, as well as between individual organisms and the gene database generated from the COG database. This was accomplished as follows. Consider two groups of CIB values, for instance, genes of *A*. *multivorum* Yenapatur with COG category P and genes from *A*. *multivorum* AIU301 that also have COG category P. Firstly, the distributions of CIB were tested for equivalence using the two-sample Anderson-Darling test [46]. The Anderson- Darling test is similar to the more familiar two- sample Kolmogorov-Smirnov test, but is generally more powerful with greater sensitivity to discrepancies in the tails of the distributions. It has null hypothesis “the two groups have the same distribution” and alternative hypothesis “the two groups have different distributions”. Secondly, if a difference was detected by the Anderson-Darling test, a further test was performed to see if the values of CIB in one group stochastically dominate those in the other group.

Stochastic dominance, also known as simple stochastic ordering or strong stochastic ordering [47], means that the probability of observing a value of CIB greater than a specified threshold in one group is always greater than the probability of seeing a value greater than the same threshold in the other group. Equivalently, one group will stochastically dominate the other if graphs of their cumulative distribution functions do not cross, though they may touch. When it applies, stochastic dominance establishes a strong relationship between two statistical samples and provides a method of comparison, in which case it can be said that one sample is stochastically smaller or larger than the other. Two groups satisfying this relationship can be ranked, say, according to their mean values, without the need to consider measures of dispersion. For the analysis in this paper, a permutation test for stochastic dominance using Monte Carlo estimation to compute the *p*-value was implemented using version 11 of the C++ programming language in conjunction with the R statistical computing software V3.3.2 (refer to S1 Appendix).

Unless otherwise indicated, the computation of CIB and all statistical analyses were carried out using the R statistical computing software V3.3.2. The kSamples package was used for the Anderson-Darling test and the Bioconductor Biostrings package was used to process DNA sequence data.

Hierarchical clustering of CIB using average linkage was carried out by means of the TM4 MeV v4.9.0 stand-alone local client using the Pearson product correlation coefficient as the distance metric [48].

## Results and discussion

### Codon usage bias in biomining organisms

Mining sites are characterized by the presence of low-pH and the prevalence of aerobic environments. These extreme conditions induce selective pressures which have an impact on indigenous organisms, for example, the principal acidophilic organisms are autotrophic, able to use ferrous iron and reduced sulfur compounds as electron donors which are released from sulfide minerals during oxidative dissolution [8]. Apart from nutritional selection, it is plausible to hypothesize that genes from such organisms have also been genetically selected in order to improve the ability of the organism to survive under extreme conditions [49].

To assess putative gene sequence differences between copper-bioleaching acidophilic species and other organisms, we considered a set of five such bacteria which inhabit the same niche (the consortium group) [25] and compared these with three specifically selected groups of bacteria (Table 1). The first group (non-consortium) includes a total of 18 previously sequenced acidophilic bacteria, which were isolated from different mining sites. The second comparison group (metagenome) is made up of 35000 sequences from a copper mine metagenomic sample including 274 bacterial families. This group is that subset of the complete metagenomic sample to which a COG category could be assigned and which had a length of at least 90% of the size of the match in the COG database. The third and final comparison group (COG), was constructed from almost all genes present in the 2014 edition of the COG database [50]. In order to make these comparisons, we used the codon information bias (CIB) as a measure of codon usage bias (see Materials and Methods).

**Table 1:** Origins of biomining strains under study.

Bacterial species	Isolation site	Reference
**Consortium**		
*Ac*. *multivorum* Yenapatur	Copper mine in the north of Chile.*	[14]
*At*. *ferrooxidans* Wenelen	Copper mine in the north of Chile.*	[14]
*At*. *thiooxidans* Licanantay	Copper mine in the north of Chile.*	[27]
*L*. *ferriphilum* Pañiwe	Copper mine in the north of Chile.*	[14]
*Sb*. *thermosulfidooxidans* Cutipay	Copper mine in the north of Chile.*	[26]
**Non-consortium**		
*A*. *ferrooxidans* ICP, DSM 10331	Hot spring run-off in Iceland.	[62]
*Ac*. *cryptum* JF-5	Coal mine lake sediment.	[32]
*Ac*. *multivorum* AIU301	pyritic acid mine drainage in the Matsuo mines.	[63]
*Ac*. sp. PM	Rio Tinto's acidic, heavy metal-rich waters.	[33]
*At*. *caldu*s ATCC 51756	Coal spoils, United Kingdom.	[34]
*At*. *caldus* SM-1	-	[64]
*At*. *ferrivorans* SS3	Norilsk mining area, Russia.	[65]
*At*. *ferrooxidans* ATCC 23270	Coal mine acid, bituminous effluent.	[66]
*At*. *ferrooxidans* ATCC 53993	-	[35]
*At*. *thiooxidans* ATCC 19377	Kimmeridge clay. Dorset, England, UK.	[67]
*D*. *acidiphilus* SJ4 DSM 22704	Sediment from acid mining effluent decantation pond; France, Chessy les Mines.	[36]
*L*. *ferriphilum* ML-04	Acidic water near a hot spring in Tengchong, Yunnan, China.	[68]
*L*. *ferrooxidans* C2-3	Volcanic ash deposit on Mount Oyama in the island of Miyake, Japan.	[37]
*Sb*. *acidophilus* TPY	Hydrothermal vent in the Pacific Ocean.	[69]
*Sb*. *thermosulfidooxidans* AT-1	Spontaneous ore deposit in Eastern Kazakhstan.	Unpublished
*Sb*. *thermosulfidooxidans* CBAR-13	Percolate solution of the bioleaching heap at Escondida copper mine, Chile.	Unpublished
*Thiomonas intermedia* K12	Sewage pipe in Hamburg Germany.	Unpublished
*Thiomonas* sp 3As	Acidic waters draining the Carnoulès mine tailings, France.	[40]
**Metagenome**		
Microbial community	Surface layer of low grade copper tailings bioleached at Dexing Copper Mine, China.	[23]

*Patent Registration No. CL 48319, Antofagasta, Chile

**Fig 1:**
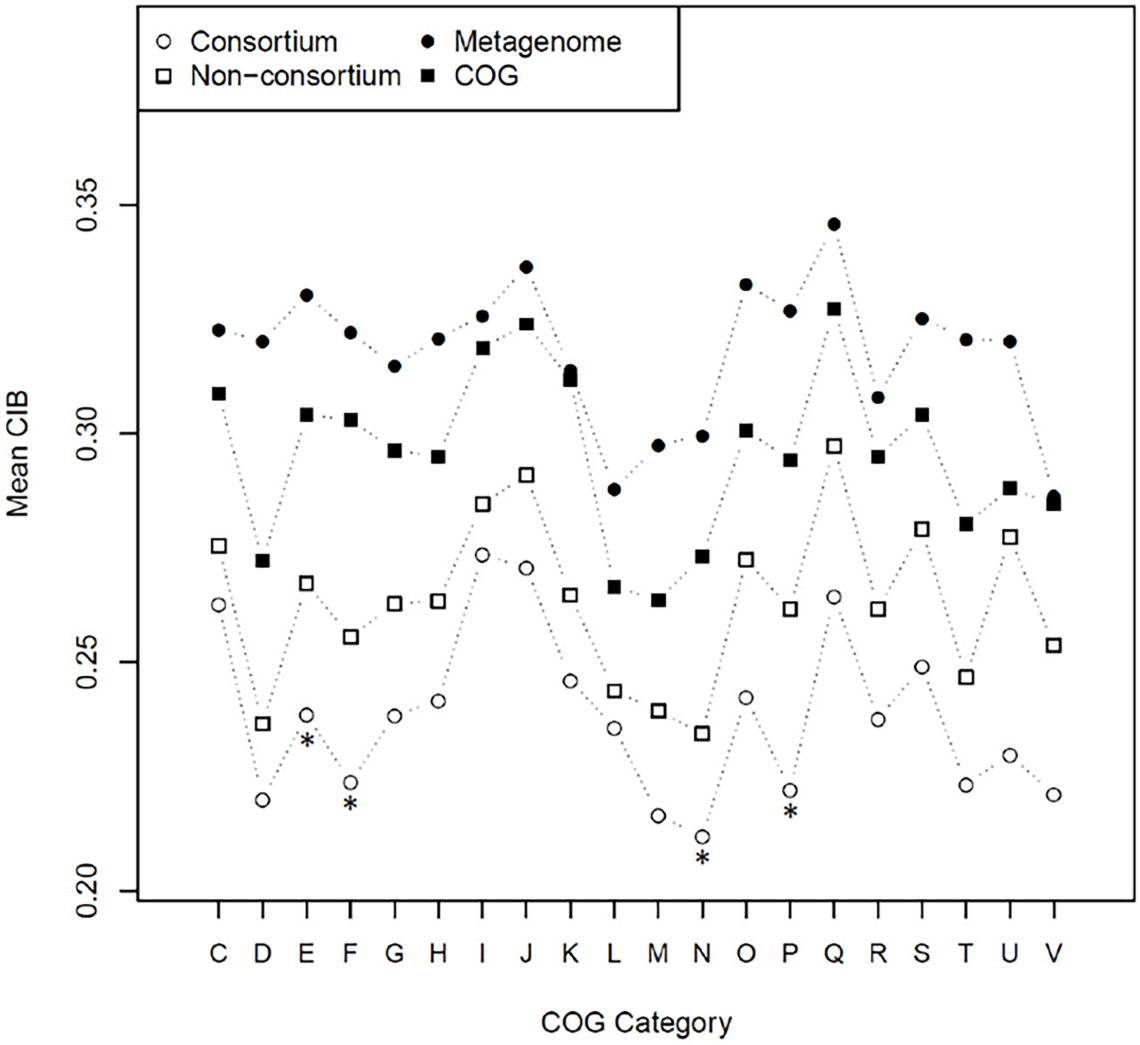
Average value of CIB for genes belonging to the consortium biomining species and the selected comparison groups under study. Each value is the average CIB calculated over all the species from each independent group classified according to COG category. The asterisks mark the four COG categories for which the greatest difference was observed between the mean CIB for the consortium and the mean CIB for the 2014 COG database.

**Fig 2:**
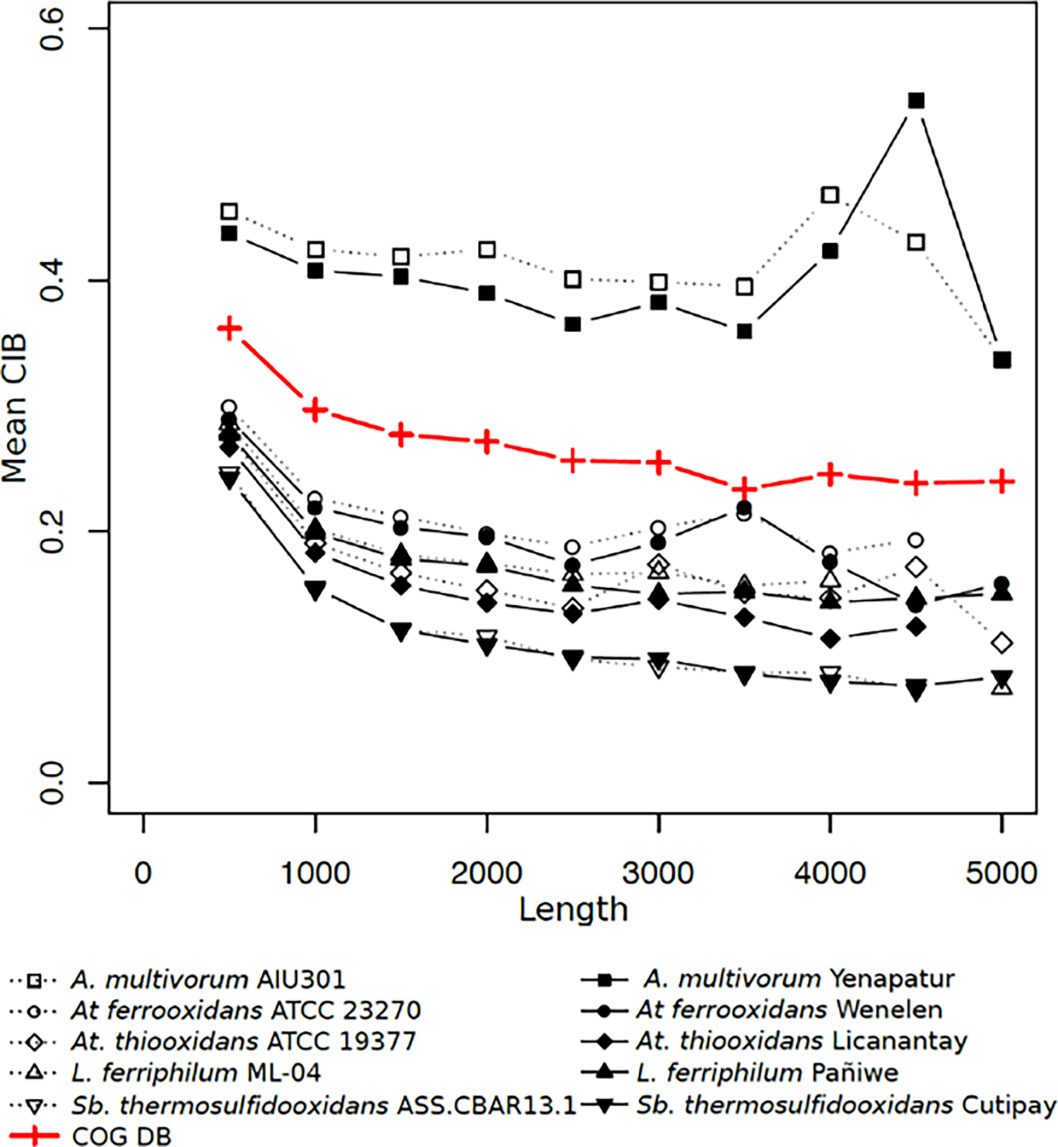
Average value of CIB for genes belonging to ten strains of bacteria (consortium and non-consortium) and the COG database binned by gene length in bases. Each bin contains genes from *ℓ*-499 to *ℓ* bases in length, where *ℓ* can be read off the x-axis. The y-axis indicates the mean value of CIB for genes belonging to the bin indicated on the x-axis. Bacterial strains belonging to the same species are plotted using the same point shape. Strains belonging to the biomining consortium are distinguished by filled points linked by solid lines while non-consortium strains are hollow points linked by dotted lines. The average CIB values for the 2014 COG database are plotted as crosses linked by a red solid line.

First, all the genes in each bacterial group were assigned COG categories. Then, the distribution of CIB values calculated for genes in each category were compared with the distribution of CIB values computed for genes in the corresponding category in the COG database. Results indicate that the consortium and non-consortium groups of acidophilic species showed significant differences in the distribution of CIB relative to the COG database in almost every category (see Fig 1 and S1 Table), with the largest differences observed in processes related to protein and nucleotide metabolism, cell motility and inorganic ion transport (COG categories E, F, N, and P). In particular, autotrophic species shown in Fig 2 (*At*. *thiooxidans*, *At*. *ferrooxidans*, *L*. *ferriphilum* and *Sb*. *thermosulfidooxidans)* exhibited smaller values of CIB on average compared to the COG database, independently of gene length.

As indicated, this is a typical characteristic found in organisms that are able to live in a wide range of habitats and which require the ability to efficiently adapt their metabolisms to different environments [51]. Also, acidophilic organisms show wide and versatile metabolic diversity, coupled with an extraordinary physiological capacity to live under extreme conditions [52]. The lower CIB seen in both acidophilic groups coincides with their capacity to adjust their metabolic variability, which correlates with previous analyses of codon usage made in other communities of microorganisms [5]. In addition, the acidophilic strains studied here are characterized by low growth rates [25], supporting the hypothesis that bacterial species with low codon usage experience slow growth [6].

All three groups of genes, (consortium, non-consortium and metagenome) exhibit distributions of CIB that differ from genes in the COG database (S1 Table). It is remarkable that genes in the first two groups have CIB values that are stochastically smaller than genes in the COG database in almost all COG categories: this is much stronger than merely saying that they have smaller CIB on average.

In contrast, while the metagenome group and COG database have different distributions of CIB, only COG categories D (cell division) and T (signal transduction mechanisms) from the metagenomics sample display a clear stochastic relationship to genes in the COG database (S1 Table), despite the mean CIB for genes in the metagenome group exceeding the mean CIB of COG database genes (Fig 1). Considering that this metagenomics sample covers a total of 274 bacterial families, it is plausible to argue that the high diversity of microorganisms in this community covers an extensive variety of patterns of CIB, which is reflected in the statistical results obtained.

### Specific codon usage bias in the acidophilic-bacteria consortium used for biomining

In order to study whether or not a higher capacity to survive in extreme environments is correlated with a particular pattern of codon usage, a fourth group of species were selected from the non-consortium group as counterpart strains to the acidophilic bacteria consortium used for biomining. This new group was composed by: *Sb*. *thermosulfidooxidans* CBAR-13, *At*. *thiooxidans* ATCC19377, *At*. *ferrooxidans* ATCC23270, *L*. *ferriphilum* ML-04 and *A*. *multivorum* AIU301, all of which were isolated from different mining sites.

In general, the observed differences in CIB are similar in both groups (Fig 2 and S2 Table), indicating that these acidophilic organisms probably share some aspects of codon usage bias independently of the place where they were isolated or their phylogenetic relationship. This is suggestive of co-evolution of the genetic code in these species [53].

In particular, the heterotrophic *A*. *multivorum* strains showed larger values of CIB on average compared to the remaining species, which are autotrophic. This was seen mainly in COG categories related to translation, transcription, signaling and general metabolism. Unlike the other members of the consortium, *A*. *multivorum* has the specific role of degrading organic metabolites highly toxic to autotrophic organisms [54]. High CIB is associated with high functional specialization and faster translational rates [55], which in this case probably improves the ability of *A*. *multivorum* to sense, metabolize and degrade organic compounds.

Unexpectedly, the low growth rate of the two *A*. *multivorum* strains [30] does not correspond to their higher CIB. However, in their extreme environmental niche (mining site), the growth of *A*. *multivorum* depends on the presence of other members of the community to produce the organic sources the bacterium consumes and to oxidize the thiosulfate compounds toxic to it [54,56]. This establishes mutual dependence within the consortium which is reflected by the similar growth rates observed in these species [56].

The next step was to divide all the genes belonging to each species into two sets, those that are conserved in the two strains of the species and those that are unique to one of the strains. For each of these sets, the two-sample Anderson-Darling test was used to decide whether or not the two strains of each species had the same distribution of CIB among the genes in each COG category.

**Fig 3:**
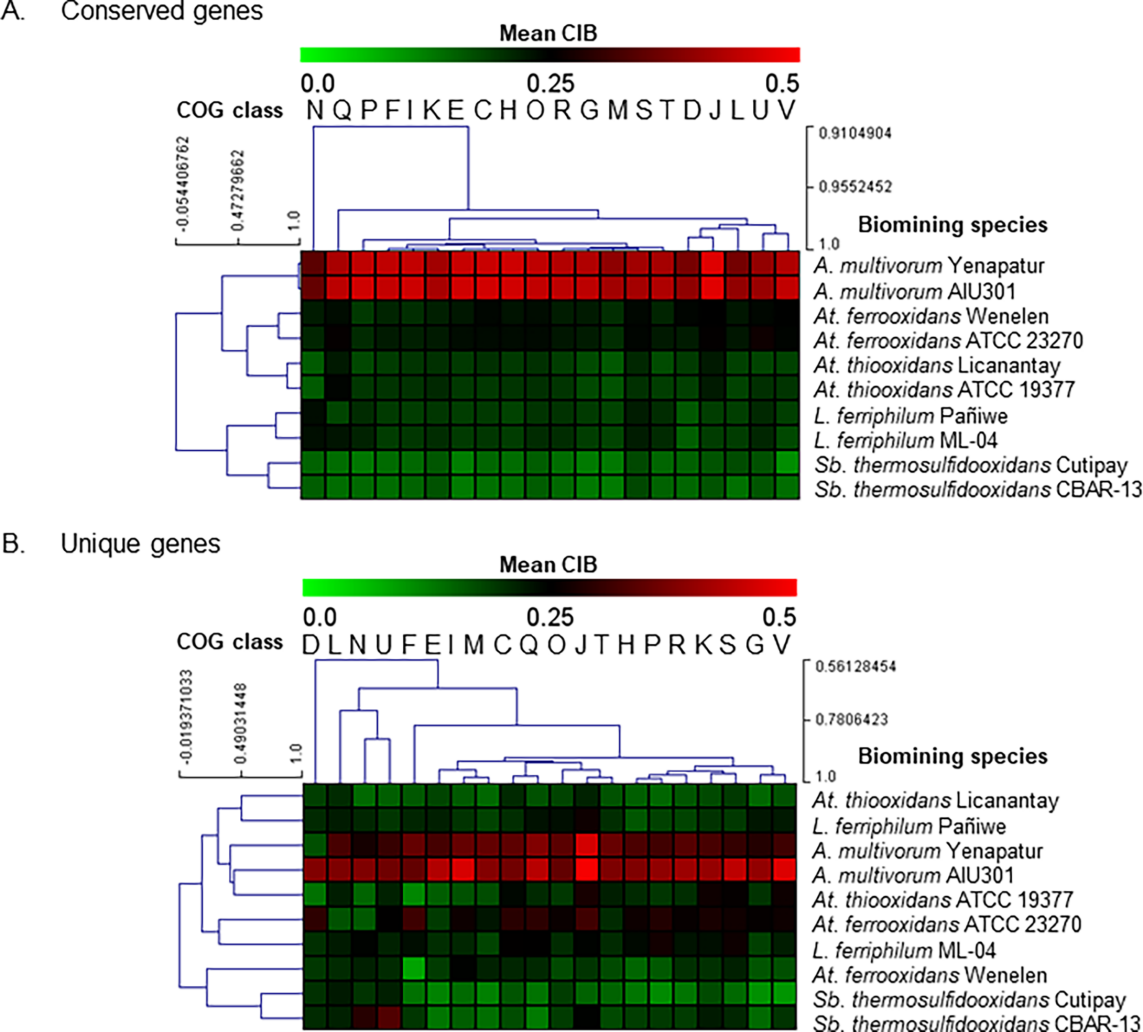
Hierarchical clustering of 10 biomining bacteria according to CIB values grouped by COG category. **A.** Conserved genes, **B.** Unique genes. For each strain, the mean value of CIB was calculated for genes (both conserved and unique) within each COG category. The strains were then hierarchically clustered using average linkage with the Pearson product correlation coefficient measuring the distance between the vectors of mean CIB per COG category. The color bar ranges from green (low CIB, 0.0) to red (high CIB, 0.5).

The results reveal that the conserved genes in all the pairs of biomining strains studied exhibit essentially the same distribution of CIB (S3 Table), supporting the previous observation that the conserved genes in biomining lifestyle organisms apparently co-evolved in order to survive in extreme environments. In addition, the clustering of conserved genes in Fig 3A shows that, biomining strains from the same species fall naturally into the same clade and hence the distribution of CIB for conserved genes among the various COG categories clusters the bacteria in a way similar to phylogenetic distance [25]. This indicates that the CIB of the conserved genes conforms to a phylogenetic relationship and has not been significantly affected by the specific niche they inhabit.

On the other hand, significant differences were identified within the group of unique genes (S4 Table). The smallest values of CIB were observed in the autotrophic species in the consortium, corresponding to low codon usage bias. Clustering based on unique genes revealed a different organization (species and COG categories) compared to clustering in terms of conserved genes (Fig 3B). Interestingly, unlike the situation observed for the conserved group of genes, consortium species *At*. *thiooxidans* Licanantay and *L*. *ferriphilum* Pañiwe (both of which stand out due to their high copper-bioleaching performance) [25], cluster together in the same clade. This supports the idea that the CIB of unique genes is most likely affected by the common niche (copper mine site) rather than any phylogenetic relationship. Note that although the two strains of *Sb*. *thermosulfidooxidans* share the same clade, both of these strains were isolated from Chilean copper deposits, which is also consistent with unique genes causing these species to be clustered according to geographic/environmental effects.

As mentioned, low CIB may suggest better environmental adaptation as a product of higher metabolic variability, which probably (directly or indirectly) affects the adaptation of the consortium to the extreme environmental conditions it inhabits. In this context, mining sites are also characterized by a high concentration of metal cations. The COG groups directly related to the adaptation to these conditions [25], such as categories L, P and V, which involve metal resistance, and iron and sulfur oxidation, presented the greatest differences between species in the consortium and their non-consortium counterparts.

Within this group of unique genes (Table 2), the following genes presented some of the lowest CIB values recorded: the protein complex TonB, which participates in iron acquisition through siderophore mechanisms [57]; the cation efflux systems, phosphate transporter and Cu-ATPase, principal proteins related to copper resistance [58,59], and the enzymes RecN and MutS which are involved in double-strand break and mismatch DNA repair respectively [60,61].

**Table 2:** Unique genes from the consortium involved in copper bioleaching with the smallest CIB values.

Gene ID	Annotation	COG	Consortium species	CIB
AFWEN_837	TonB-dependent receptor	P	*At*. *ferrooxidans* Wenelen	0.045
AFWEN_932	Iron complex outermembrane recepter protein	P	*At*. *ferrooxidans* Wenelen	0.069
ATLIC_1742	Cation efflux system protein	P	*At*. *thiooxidans* Licanantay	0.076
ATLIC_1133	TonB-dependent receptor	P	*At*. *thiooxidans* Licanantay	0.078
ATLIC_2149	Iron complex outermembrane recepter protein	P	*At*. *thiooxidans* Licanantay	0.085
ATLIC_3279	ATPase-like protein	L	*At*. *thiooxidans* Licanantay	0.085
ATLIC_1945	superfamily I DNA helicase	L	*At*. *thiooxidans* Licanantay	0.085
ATLIC_223	DNA mismatch repair protein MutS domain protein	L	*At*. *thiooxidans* Licanantay	0.089
ATLIC_3543	TonB-dependent receptor	P	*At*. *thiooxidans* Licanantay	0.089
ATLIC_3440	Iron complex outermembrane recepter protein	P	*At*. *thiooxidans* Licanantay	0.104
ATLIC_3544	Iron complex outermembrane recepter protein	P	*At*. *thiooxidans* Licanantay	0.105
ATLIC_3112	Phosphate transporter	P	*At*. *thiooxidans* Licanantay	0.113
ATLIC_3827	Cu2+-exporting ATPase	P	*At*. *thiooxidans* Licanantay	0.118
ATLIC_3828	Cu2+-exporting ATPase	P	*At*. *thiooxidans* Licanantay	0.143
ATLIC_3862	Cu(I)/Ag(I) efflux system membrane protein CusA	V	*At*. *thiooxidans* Licanantay	0.160
LFPA_2449	Heavy-metal exporter, HME family	P	*L*. *ferriphilum* Pañiwe	0.120
LFPA_613	Cu2+-exporting ATPase	P	*L*. *ferriphilum* Pañiwe	0.169
STCUT_2973	Major facilitator superfamily MFS_1	P	*Sb*. *thermosulfidooxidans* Cutipay	0.062
STCUT_3247	Putative efflux protein	P	*Sb*. *thermosulfidooxidans* Cutipay	0.077
STCUT_3508	Putative efflux protein	P	*Sb*. *thermosulfidooxidans* Cutipay	0.077
STCUT_2348	DNA repair protein RecN (Recombination protein N)	L	*Sb*. *thermosulfidooxidans* Cutipay	0.093
STCUT_1513	ATP-dependent DNA helicase RecQ	L	*Sb*. *thermosulfidooxidans* Cutipay	0.115
STCUT_1001	Cation efflux system protein, CDF family	P	*Sb*. *thermosulfidooxidans* Cutipay	0.158

One of the principal characteristics of the acidophilic consortium used for bioleaching in comparison to other acidophilic organisms, is the ability of its members to resist elevated concentrations of heavy metal cations [25]. Most of the consortium species resist at least twice the external copper concentration in relation to their counterpart biomining species (S5 Table). Next, a collection of genes previously classified in copper resistance and oxidative stress protection were selected from the acidophilic consortium [25], together with homologs from their non-consortium counterparts. Inside this group, copper efflux proteins CopA and Cus, antioxidant defense enzymes thioredoxin reductase (NadpH), superoxide dismutase (sodA) and peroxidereoxin (bcp) components exhibit lower CIB values in relation to the homologous genes of their acidophilic counterpart species (Table 3). The species belonging to the consortium not only contain a higher number of these components [25], but these genes also present with a low value of CIB, suggesting higher transcriptional adaptation to living in a wide range of habitats, which was probably acquired as a measure for resisting the extreme, elevated copper conditions present in the mine.

**Table 3:** List of genes from the consortium with lower CIB values compared to its non-consortium counterpart.

Id consortium	CIB consortium	Id counterpart	CIB counterpart	name	Gene annotation	COG
**Copper resistance**						
LFPA_558	0.254	LFML04_RS04475	0.257	cutA	periplasmic divalent cation tolerance protein	P
ATLIC_3492	0.142	ATHIO_RS0103355	0.145		Cu2+-exporting ATPase	P
STCUT_215	0.069	AOA63_RS11310	0.074	copA	heavy metal translocating P-type ATPase	P
STCUT_220	0.093	AOA63_RS11335	0.100		Cu2+-exporting ATPase	P
LFPA_1158	0.208	LFML04_RS01790	0.217		Cu2+-exporting ATPase	P
ATLIC_2000	0.245	ATHIO_RS0110590	0.254		lipoprotein	M,P
ATLIC_1679	0.288	ATHIO_RS0112045	0.299	hmrR	transcriptional regulator, MerR family	K
STCUT_1350	0.158	AOA63_RS16975	0.196		NIF3-related protein	S
AFWEN_2071	0.231	AFE_RS11165	0.278	cusA	Cu(I)/Ag(I) efflux system membrane protein CusA	V
LFPA_601	0.148	LFML04_RS03050	0.216	cusA	Cu(I)/Ag(I) efflux system membrane protein CusA	V
LFPA_601	0.148	LFML04_RS08675	0.224	cusA	Cu(I)/Ag(I) efflux system membrane protein CusA	V
AMYEN_2455	0.250	ACMV_RS13835	0.521		Cu2+-exporting ATPase	P
**Average**	**0.186**		**0.232**			
**ROS**						
ATLIC_1147	0.332	ATHIO_RS0106580	0.333	sodA	superoxide dismutase, Fe-Mn family	P
ATLIC_1534	0.188	ATHIO_RS0115785	0.196		peroxidase (EC:1.11.1.7)	O
LFPA_122	0.167	LFML04_RS12970	0.176	resA	thiol-disulfide oxidoreductase	O,C
LFPA_382	0.254	LFML04_RS05360	0.264	trxA	thioredoxin	O,C
ATLIC_2542	0.304	ATHIO_RS0105865	0.314		alkylhydroperoxidase like protein, AhpD family	S
LFPA_1163	0.227	LFML04_RS01815	0.237		thioredoxin 2	O,C
STCUT_347	0.284	AOA63_RS12055	0.295		alkylhydroperoxidase like protein, AhpD family	S
AFWEN_3145	0.259	AFE_RS01735	0.271		glutaredoxin family protein	O
AMYEN_133	0.392	ACMV_RS07445	0.406	trxA	thioredoxin 1	O,C
ATLIC_1449	0.140	ATHIO_RS0107230	0.155	msrA	peptide-methionine (S)-S-oxide reductase	O
AMYEN_129	0.305	ACMV_RS07425	0.323	trxB	thioredoxin reductase (NADPH)	O
ATLIC_1450	0.183	ATHIO_RS0107225	0.204	msrB	peptide-methionine (R)-S-oxide reductase	O
AFWEN_2550	0.162	AFE_RS06860	0.184	bcp	peroxiredoxin Q/BCP	O
ATLIC_2479	0.201	ATHIO_RS0106370	0.227	bcp	peroxiredoxin Q/BCP	O
ATLIC_130	0.240	ATHIO_RS0108025	0.272	ahpC	peroxiredoxin (alkyl hydroperoxide reductase subunit C)	O
STCUT_838	0.130	AOA63_RS14385	0.163		glutaredoxin-like domain-containing protein	O
AFWEN_1580	0.267	AFE_RS13515	0.300	msrA	peptide-methionine (S)-S-oxide reductase	O
STCUT_1306	0.178	AOA63_RS16755	0.223	sodA	superoxide dismutase, Fe-Mn family	P
LFPA_1179	0.304	LFML04_RS01895	0.351	bcp	peroxiredoxin Q/BCP	O
STCUT_518	0.252	AOA63_RS12875	0.317	bcp	peroxiredoxin Q/BCP	O
LFPA_1314	0.244	LFML04_RS02930	0.316		monothiol glutaredoxin	O
AFWEN_3137	0.162	AFE_RS01775	0.234	trxB	thioredoxin reductase (NADPH)	O
ATLIC_589	0.246	ATHIO_RS0110035	0.319		monothiol glutaredoxin	O
**Average**	**0.236**		**0.264**			

## Conclusions

In general, acidophilic organisms present similar patterns of CIB independently of the place where they were isolated or their phylogenetic relationships and this is mainly characterized by low CIB in autotrophic species. However, the particular copper mining environment influences the CIB in unique genes and genes for copper-resistance, probably conferring the acidophilic consortium with a greater capacity to resist high concentrations of metal cations. Finally, studies of CIB in acidophilic organisms provide an alternative application for identifying and characterizing new strains with higher capacities for bioleaching metal ores.

## Supporting information

S1 Table Summary of statistical relationships between the CIB distribution in each group under study and genes from the COG2014 database for each COG category. Each table entry displays the p-value from the permutation test for stochastic dominance between genes from the indicated group and COG category and genes assigned the same COG category in the COG database.
(PDF)

S2 Table Summary of statistically significant differences in CIB distribution between biomining strains (the consortium species and their non-consortium counterparts) and genes from the COG2014 database for each COG category. Each table entry displays the difference in the mean value of CIB for genes of the indicated strain and COG category, and the mean value of CIB for genes assigned the same COG category in the COG database.
(PDF)

S3 Table Statistically significant differences in CIB distribution of conserved genes between the consortium strain and its counterpart for each species and COG category. Each table entry displays the p-value for the two-sample Anderson-Darling test applied to genes conserved in the consortium and non-consortium strains for the indicated species and COG category.
(PDF)

S4 Table Statistically significant differences in CIB distribution of unique genes between the consortium strain and its non-consortium counterpart for each species and COG category. Each table entry displays the p-value for the two-sample Anderson-Darling test applied to unique genes in the consortium and non-consortium strains for the indicated species and COG category.
(PDF)

S5 Table Minimum inhibitory concentration (MIC) of copper between each consortium strain and its non-consortium counterpart. *data from [25].
(PDF)

S1 Appendix Kullback–Leibler codon information bias (CIB) and stochastic dominance. (PDF)
