# Supplementary material for: Codon usage bias reveals genomic adaptations to environmental conditions in an acidophilic consortium
Source: PLoS One. 2018 May 9;13(5):e0195869. doi: 10.1371/journal.pone.0195869 (PMC5942774; doi:10.1371/journal.pone.0195869)
Supplement: S1 Table — Each table entry displays the p-value from the permutation test for stochastic dominance between genes from the indicated group and COG category and genes assigned the same COG category in the COG database. (PDF) [file pone.0195869.s001.pdf]

S1 Table. Summary of statistical relationships between the CIB distribution in each group under study and genes from the COG2014 database for each COG category. Each table entry displays the p-value from the permutation test for stochastic dominance between genes from the indicated group and COG category and genes assigned the same COG category in the COG database.

COG Class	Consortium	Non-consortium	Metagenome
C	0.007*<	0.017*<	0.513*
D	0.028*<	0.017*<	0.026*>
E	0.006*<	0.000*<	0.376*
F	0.008*<	0.001*<	0.443*
G	0.003*<	0.001*<	0.422*
H	0.008*<	0.003*<	0.298*
I	0.011*<	0.002*<	0.731*
J	0.009*<	0.003*<	0.344*
K	0.001*<	0.001*<	0.898*
L	0.206*	0.032*<	0.331*
M	0.024*<	0.019*<	0.136*
N	0.001*<	0.001*<	0.201*
O	0.001*<	0.002*<	0.084*
P	0.001*<	0.000*<	0.273*
Q	0.005*<	0.017*<	0.405*
R	0.001*<	0.006*<	0.453*
S	0.000*<	0.002*<	0.166*
T	0.031*<	0.007*<	0.036*>
U	0.001*<	0.645*	0.308*
V	0.003*<	0.006*<	0.945*

* The p-value from the two-sample Anderson-Darling test (not shown in the table) indicates a statistically significant difference inn the distribution of CIB for genes from the indicated group and COG category compared to genes from the same COG category in the COG database.

< The p-value from the permutation test for stochastic dominance (shown), together with the sign of the test statistic (not shown), indicates that the CIB for genes in the indicated group and COG category is stochastically smaller than the CIB of genes of the same COG category in the COG database.

> The p-value from the permutation test for stochastic dominance (shown), together with the sign of the test statistic (not shown), indicates that the CIB for genes in the indicated group and COG category is stochastically larger than the CIB of genes of the same COG category in the COG database.

NOTE: the p-values in the table have been adjusted for multiple testing using the Benjamini-Hochberg procedure (FDR).
