# Supplementary material for: Codon usage bias reveals genomic adaptations to environmental conditions in an acidophilic consortium
Source: PLoS One. 2018 May 9;13(5):e0195869. doi: 10.1371/journal.pone.0195869 (PMC5942774; doi:10.1371/journal.pone.0195869)
Supplement: S2 Table — Each table entry displays the difference in the mean value of CIB for genes of the indicated strain and COG category, and the mean value of CIB for genes assigned the same COG category in the COG database. (PDF) [file pone.0195869.s002.pdf]

S2 Table. Summary of statistically significant differences in CIB distribution between biomining strains (the consortium species and their non-consortium counterparts) and genes from the COG2014 database for each COG category. Each table entry displays the difference in the mean value of CIB for genes of the indicated strain and COG category, and the mean value of CIB for genes assigned the same COG category in the COG database.

Consortium strains

**COG class**	***A. multivorum*** **Yenapatur**	***At. ferrooxidans*** **Wenelen**	***At. thiooxidans*** **Licanantay**	***L. ferriphilum*** **Pañiwe**	***Sb. thermosulfidooxidans*** **Cutipay**
C	+0.127*	-0.068*	-0.104*	-0.113*	-0.156*
D	+0.065*	-0.041*	-0.081*	-0.099*	-0.100*
E	+0.119*	-0.081*	-0.113*	-0.110*	-0.179*
F	+0.125*	-0.101*	-0.120*	-0.108*	-0.160*
G	+0.108*	-0.080*	-0.127*	-0.101*	-0.174*
H	+0.128*	-0.067*	-0.107*	-0.112*	-0.154*
I	+0.116*	-0.108*	-0.129*	-0.127*	-0.171*
J	+0.145*	-0.075*	-0.100*	-0.126*	-0.157*
K	+0.081*	-0.097*	-0.118*	-0.114*	-0.143*
L	+0.077*	-0.047*	-0.065*	-0.063*	-0.062*
M	+0.121*	-0.057*	-0.105*	-0.080*	-0.133*
N	+0.058*	-0.052	-0.118*	-0.042*	-0.110*
O	+0.128*	-0.071*	-0.106*	-0.097*	-0.136*
P	+0.122*	-0.107*	-0.124*	-0.091*	-0.167*
Q	+0.082*	-0.093*	-0.115*	-0.140*	-0.187*
R	+0.116*	-0.077*	-0.112*	-0.098*	-0.154*
S	+0.087*	-0.066*	-0.100*	-0.089*	-0.135*
T	+0.117*	-0.075*	-0.107*	-0.074*	-0.129*
U	+0.087*	-0.057*	-0.117*	-0.082*	-0.108*
V	+0.109*	-0.056*	-0.105*	-0.083*	-0.176*

continuation supplementary table 2

Non-consortium counterpart strains

COG class	*A. multivorum* AIU301	*At. ferrooxidans* ATCC 23270	*At. thiooxidans* ATCC 19377	*L. ferriphilum* ML-04	*Sb. thermosulfidooxidans* CBAR-13
C	+0.139*	-0.065*	-0.110*	-0.106*	-0.165*
D	+0.117*	-0.043*	-0.089*	-0.108*	-0.113*
E	+0.148*	-0.069*	-0.114*	-0.111*	-0.174*
F	+0.133*	-0.077*	-0.105*	-0.108*	-0.155*
G	+0.136*	-0.072*	-0.116*	-0.107*	-0.162*
H	+0.152*	-0.055*	-0.096*	-0.098*	-0.147*
I	+0.134*	-0.096*	-0.122*	-0.121*	-0.166*
J	+0.150*	-0.064*	-0.096*	-0.124*	-0.147*
K	+0.101*	-0.078*	-0.085*	-0.096*	-0.131*
L	+0.125*	-0.048*	-0.074*	-0.068*	-0.108*
M	+0.142*	-0.057*	-0.094*	-0.080*	-0.132*
N	+0.080*	-0.064*	-0.114*	-0.036*	-0.098*
O	+0.123*	-0.057*	-0.099*	-0.093*	-0.138*
P	+0.152*	-0.080*	-0.098*	-0.067*	-0.136*
Q	+0.107*	-0.063*	-0.088*	-0.095*	-0.185*
R	+0.134*	-0.062*	-0.095*	-0.082*	-0.140*
S	+0.116*	-0.052*	-0.082*	-0.061*	-0.114*
T	+0.133*	-0.057*	-0.086*	-0.072*	-0.122*
U	+0.102*	-0.025*	-0.081*	-0.074*	-0.089*
V	+0.162*	-0.033*	-0.062*	-0.093*	-0.118*

* The p-value from the two-sample Anderson-Darling test (not shown) indicates a Statistically significant difference inn the distribution of CIB for genes of the indicated organism and COG category compared to genes of the same COG category in the COG database.

NOTE: for each subtable, the p-values were adjusted for multiple testing using the Benjamini-Hochberg procedure (FDR).
