# Supplementary material for: Codon usage bias reveals genomic adaptations to environmental conditions in an acidophilic consortium
Source: PLoS One. 2018 May 9;13(5):e0195869. doi: 10.1371/journal.pone.0195869 (PMC5942774; doi:10.1371/journal.pone.0195869)
Supplement: S3 Table — Each table entry displays the p-value for the two-sample Anderson-Darling test applied to genes conserved in the consortium and non-consortium strains for the indicated species and COG category. (PDF) [file pone.0195869.s003.pdf]

S3 Table. Statistically significant differences in CIB distribution of conserved genes between the consortium strain and its counterpart for each species and COG category. Each table entry displays the p-value for the two-sample Anderson-Darling test applied to genes conserved in the consortium and non-consortium strains for the indicated species and COG category.

COG class	*A. multivorum*	*At. ferrooxidans*	*At. thiooxidans*	*L. ferriphilum*	*Sb. thermosulfidooxidans*
C	1.000	1.000	1.000	1.000	1.000
D	1.000	1.000	1.000	1.000	1.000
E	1.000	1.000	1.000	1.000	1.000
F	1.000	1.000	1.000	1.000	1.000
G	1.000	1.000	1.000	1.000	1.000
H	1.000	1.000	1.000	1.000	1.000
I	1.000	1.000	1.000	1.000	1.000
J	1.000	1.000	1.000	1.000	1.000
K	1.000	1.000	1.000	1.000	1.000
L	1.000	1.000	1.000	1.000	0.047*
M	1.000	1.000	1.000	1.000	1.000
N	1.000	1.000	1.000	1.000	1.000
O	1.000	1.000	1.000	1.000	1.000
P	1.000	1.000	1.000	1.000	1.000
Q	1.000	1.000	1.000	1.000	1.000
R	1.000	1.000	1.000	1.000	1.000
S	1.000	1.000	1.000	1.000	1.000
T	1.000	1.000	1.000	1.000	1.000
U	1.000	1.000	1.000	1.000	1.000
V	1.000	1.000	1.000	1.000	0.428

* The p-value from the two-sample Anderson-Darling test indicates a statistically significant difference in the distribution of CIB for unique genes from the two strains of the indicated

NOTE: the p-values in the table have been adjusted for multiple testing using the Benjamini-Hochberg procedure (FDR).
