# Supplementary material for: Codon usage bias reveals genomic adaptations to environmental conditions in an acidophilic consortium
Source: PLoS One. 2018 May 9;13(5):e0195869. doi: 10.1371/journal.pone.0195869 (PMC5942774; doi:10.1371/journal.pone.0195869)
Supplement: S4 Table — Each table entry displays the p-value for the two-sample Anderson-Darling test applied to unique genes in the consortium and non-consortium strains for the indicated species and COG category. (PDF) [file pone.0195869.s004.pdf]

S4 Table . Statistically significant differences in CIB distribution of unique genes between the consortium strain and its non-consortium counterpart for each species and COG category. Each table entry displays the p-value for the two-sample Anderson-Darling test applied to unique genes in the consortium and non-consortium strains for the indicated species and COG category.

COG class	*A. multivorum*	*At. ferrooxidans*	*At. thiooxidans*	*L. ferriphilum*	*Sb. thermosulfidooxidans*
C	0.905	0.206	0.377	0.163	0.943
D	0.064	0.146	0.485	0.943	0.757
E	0.022*	0.943	0.476	0.515	0.343
F	0.969	0.176	0.638	0.810	0.347
G	0.021*	0.091	0.515	0.203	0.001*
H	0.176	0.206	0.476	0.216	0.009*
I	0.072	0.905	0.573	0.942	0.905
J	0.881	0.216	0.203	0.515	0.026l*
K	0.026*	0.058	0.000*	0.176	0.043*
L	0.172	0.050*	0.757	0.969	0.377
M	0.258	0.671	0.342	0.969	0.515
N	0.638	0.881	0.861	0.485	0.210
O	0.711	0.163	0.711	0.853	0.964
P	0.066	0.009*	0.025*	0.021*	0.000*
Q	0.556	0.460	0.758	0.706	0.905
R	0.061	0.021*	*0.026	0.018*	0.003*
S	0.000*	0.172	0.001*	0.061	0.003*
T	0.387	0.330	0.079	0.670	0.176
U	0.401	0.314	0.061	0.313	0.058
V	0.027*	0.021*	0.006*	0.943	0.009*

* The p-value from the two-sample Anderson-Darling test indicates a statistically significant difference inn the distribution of CIB for unique genes from the two strains of the indicated species and COG category.

NOTE: the p-values in the table have been adjusted for multiple testing using the Benjamini-Hochberg procedure (FDR).
