# Supplementary material for: Codon usage bias reveals genomic adaptations to environmental conditions in an acidophilic consortium
Source: PLoS One. 2018 May 9;13(5):e0195869. doi: 10.1371/journal.pone.0195869 (PMC5942774; doi:10.1371/journal.pone.0195869)
Supplement: S5 Table — *data from [25]. (PDF) [file pone.0195869.s005.pdf]

S5 Table. Minimum inhibitory concentration (MIC) of copper between each consortium strain and its non-consortium counterpart. *data from [25].

	MIC	
**Consortium**	**20 °C**	**30 °C**
At. ferrooxidans Wenelen	>10	>10
At. thiooxidans Licanantay	8	8
A. multivorum Yenapatur	2	2
L. ferriphilum Pañiwue	5	5
Sb thermosulfidooxidans Cutipay	n.d.	n.d.

	MIC	
**Counterpart**	**20 °C**	**30 °C**
At. ferrooxidans ATCC23270	5	55
At. thiooxidans ATCC19377	n.d.	n.d.
A. multivorum AIU301	0,1	0,2
L. ferriphilum ML-04	n.d.	< 3
Sb. thermosulfidooxidans CBAR-13	n.d.	n.d.
