# Supplementary material for: Codon usage bias reveals genomic adaptations to environmental conditions in an acidophilic consortium
Source: PLoS One. 2018 May 9;13(5):e0195869. doi: 10.1371/journal.pone.0195869 (PMC5942774; doi:10.1371/journal.pone.0195869)
Supplement: S1 Appendix — (PDF) [file pone.0195869.s006.pdf]

SUPPLEMENTARY MATERIAL

1. *Kullback–Leibler codon information bias (CIB)*

The Kullback-Leibler codon information bias (CIB) is a measure of codon usage bias (CUB) based on information theoretic concepts which takes account of how amino acids are distributed (see [44], for an examination of various measures of CUB based on other principles such as the effective number of codons, scaled χ^2, codon bias index, etc.). As an aside, note that GC3s, GC% calculated over the third position of each codon in a coding sequence, is itself a simple measure of codon usage bias. However, GC3s does not account for the structure of synonymous families of codons engendered in the genetic code and hence does not necessarily portray codon usage bias in the same way as measures like CIB. CIB was defined in [45] as follows:

$$\Delta = \sum_{a \in \mathcal{A}} q_a \log|a| - \sum_{a \in \mathcal{A}} q_a \left(\sum_{i \in a} \frac{p_i}{q_a} \log \frac{p_i}{q_a} \right),$$

where p_i is the relative frequency of codon i,

$$q_a = \sum_{i \in a} p_i$$

is the relative frequency of amino acid a and $\mathcal{A}$ represents the appropriate genetic code,

that is, $\mathcal{A}$ gives the mapping between amino acids and codons. More precisely, each amino acid a in $\mathcal{A}$ is represented as the set of codons that code for it, for example, if a is alanine (Ala), then a is the set {GCA,GCG,GCT,GCC}. Finally, $|a|$ denotes the number of codons that code for amino acid a, for example, $|a|=4$ if a is alanine.

Being based on entropy, CIB is a natural quantity for measuring the departure of a coding sequence from equal usage of synonymous codons. It is zero if and only if the codons that code for each amino acid are used equally often to represent that amino acid, that is, there is unbiased synonymous codon

usage. The maximum value that Δ can take is $\sum_{a\in\mathcal{A}} q_a \log|a|$, which is attained precisely when each amino acid is represented by exactly one codon. Smaller values of CIB correspond to low (less selective or weak) CUB while larger values of CIB correspond to a greater concentration of the codon relative frequencies on fewer codons (stronger or more selective CUB). Finally, the appearance of the relative frequencies q_a of the amino acids in the formula for Δ allows CIB to account for differences in amino-acid distribution so that CIB can be used to compare the strength of CUB in coding sequences from different sources, for example, genes in the same organism or orthologs in different organisms.

For this study, Δ was rescaled to have a value in the range 0–1 as follows:

$$\underline{\Delta}=\frac{\Delta}{\sum_{a\in\mathcal{A}} q_a \log|a|}=1-\frac{\sum_{a\in\mathcal{A}} q_a \left(\sum_{i\in a} \frac{p_i}{q_a}\log\frac{p_i}{q_a}\right)}{\sum_{a\in\mathcal{A}} q_a \log|a|}.$$

Thus, $\underline{\Delta}$=0 corresponds to no codon usage bias (the same as Δ=0), but $\underline{\Delta}$=1 always corresponds to the most extremely selective case of CUB in which each amino acid is represented by exactly one codon.

2. *Stochastic Dominance*

The test statistic used in the permutation test for stochastic dominance was

¡Error!

where *F* and *G* denote the empirical distribution functions estimated for the two groups. The statistic *S* takes values in the range −1 to 1, values near 1 indicating that the second group stochastically dominates the first group, values near −1 signifying the converse and intermediate values resulting when there is

no clear stochastic relationship. The test for stochastic dominance has hypotheses, H_0 there is no stochastic dominance relationship between the two groups H_a: one of the groups stochastically dominates the other. All *p*- values reported for the stochastic dominance test were estimated using 10000 Monte Carlo replicates.
